# Native qudit entanglement in a trapped ion quantum processor

**DOI:** 10.1038/s41467-023-37375-2

**Published:** 2023-04-19

**Authors:** Pavel Hrmo, Benjamin Wilhelm, Lukas Gerster, Martin W. van Mourik, Marcus Huber, Rainer Blatt, Philipp Schindler, Thomas Monz, Martin Ringbauer

**Affiliations:** 1 Institut für Experimentalphysik, Universität Innsbruck, Technikerstraße 25/4, 6020 Innsbruck, Austria; 2 Atominstitut, Technische Universität Wien, 1020 Vienna, Austria; 3 Institute for Quantum Optics and Quantum Information-IQOQI Vienna, Austrian Academy of Sciences, Boltzmanngasse 3, 1090 Vienna, Austria; 4 Institut für Quantenoptik und Quanteninformation, Österreichische Akademie der Wissenschaften, Technikerstraße 21a, 6020 Innsbruck, Austria; 5 AQT, Technikerstraße 17, 6020 Innsbruck, Austria

**Keywords:** Quantum information, Qubits

## Abstract

Quantum information carriers, just like most physical systems, naturally occupy high-dimensional Hilbert spaces. Instead of restricting them to a two-level subspace, these high-dimensional (qudit) quantum systems are emerging as a powerful resource for the next generation of quantum processors. Yet harnessing the potential of these systems requires efficient ways of generating the desired interaction between them. Here, we experimentally demonstrate an implementation of a native two-qudit entangling gate up to dimension 5 in a trapped-ion system. This is achieved by generalizing a recently proposed light-shift gate mechanism to generate genuine qudit entanglement in a single application of the gate. The gate seamlessly adapts to the local dimension of the system with a calibration overhead that is independent of the dimension.

## Abstract (web summary)

Encoding quantum information in qudits instead of qubits allows for several advantages, but scalable native entangling techniques would be needed. Here, the authors show how to use light-shift gates to perform entangling operations on trapped ion systems, with a calibration overhead which is independent on the qudit dimension.

## Introduction

**Fig. 1:**
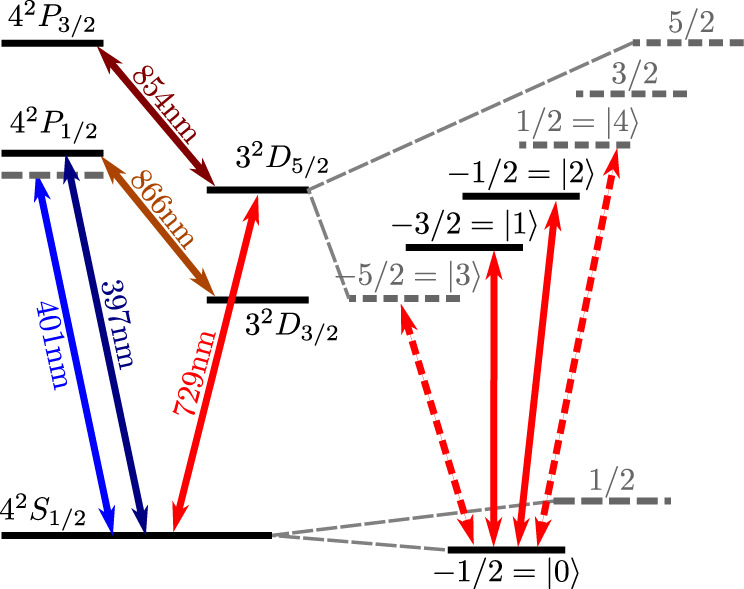
Level scheme of the ^40^Ca^+^ ion. Encoding quantum information in the sub-levels of the *S*_1/2_ and *D*_5/2_ manifold allows us to increase the size of the computational Hilbert space. Coherent operations between the sub-levels can be performed using 729 nm laser light, while 401 nm light is used to generate the light shift for the state-dependent force.

Quantum computing has taken great strides in the past decades with multiple platforms demonstrating control over tens of qubits^1–4^. However, scaling these systems to a regime beyond the capabilities of classical computers remains very challenging, both in terms of increasing the size of the quantum computational Hilbert space, and in terms of increasing the depth of the computational circuits. A significant potential for tempering these daunting scaling demands, however, lies in plain sight when appreciating that the quantum systems we use are multi-level, not two-level systems. A natural way to extend the computational Hilbert space and reduce circuit complexity, without increasing the complexity of quantum devices is thus to use the full multi-level or qudit structure of existing quantum information carriers such as trapped ions, see Fig. 1.

Qudit control has already been demonstrated in a number of architectures^5–16^, including trapped ions^17–20^. Qudit approaches not only enable reduced circuit complexity^21^ and simplifications of virtually any quantum circuit^22^, but also benefit from more powerful quantum error correction^23–26^, and enable the implementation of optimal quantum measurements^27^ as well as native quantum simulation of a range of physical systems such as lattice gauge models^28,29^, optimization problems^30^, or quantum chemistry^31^. The key to these advantages, however, is an appropriate set of quantum operations. While for qubits all entangling gates are equal up to local rotations, the same is not true for qudits, where the richer Hilbert space allows for different forms of coherence^6^ and entanglement^32^. We can roughly classify qudit entangling gates by their entangling power, i.e., the amount of entanglement that is created relative to a maximally entangled state of two qubits. While in principle already the simplest gate, a qubit entangling gate embedded in a higher-dimensional Hilbert space, would suffice for universal qudit quantum computation^17,33^, having access to a range of gates with different entangling power will be crucial for unlocking the full potential of a qudit quantum processor. Not only will a diverse set of interactions enable the direct simulation of a wider range of physical systems, but it will also enable much more efficient quantum circuit decomposition.

Here we describe and demonstrate a native qudit entangling gate in a trapped-ion quantum processor. Being based on differential light shifts between the ground- and excited state manifolds on an optical transition, the gate action can be made symmetric on all excited qudit states. We show that this implies that the same gate mechanism can be used irrespective of the qudit dimension to generate genuine qudit entanglement in the sense that the Schmidt number of the resulting state equals the qudit dimension. Crucially, this means that the experimental calibration overhead does not increase with qudit dimension. We further show that direct application of the gate can generate maximal qudit entanglement up to dimension 4. We characterize the gate dynamics and noise sources in detail, demonstrating that this gate can be a stepping stone into the world of native qudit quantum information processing with trapped ions.

## Results

**Fig. 2:**
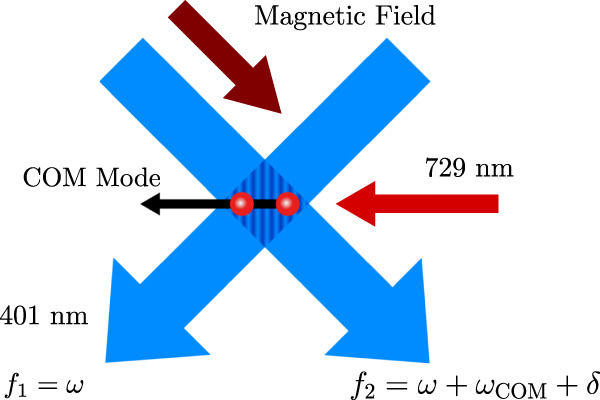
Experimental setup. Two beams with *λ* = 401 nm and frequencies *f*_1_,  *f*_2_ with a relative detuning of *ω*_COM_ + *δ* are intersected at a 90^∘^ angle to drive the state-dependent force. A 729 nm laser along the axial trap direction is used to implement local operations.

The principle behind the qudit entanglement generation is the application of light-shift (LS) gates, in which a state-dependent optical-dipole force couples the ions’ electronic states to their common motion in the trap. Light-shift gates have been well studied for entangling hyperfine and Zeeman qubits with a pair of intersecting laser beams (see Fig. 2) that create a traveling wave^34–37^. The traveling wave produces a spatially modulated light shift that drives an excursion in the phase space of one of the motional modes. The ions acquire a different geometric phase depending on their electronic state, leaving them in an entangled state after completing the excursion. The beams also introduce additional, unwanted local phases as the electronic states experience different light shifts. For hyperfine or Zeeman qubits, the polarization of the optical beams can be used to null these differential light shifts on the electronic states. In practice, however, these gates are typically applied with a spin echo pulse, inserted between two halves of the LS gate pulse during which the ions complete a single loop in motional phase space. This ensures that any residual light shifts between the two electronic states due to experimental imperfections are canceled. Adding these local *π* pulses has the further advantage of suppressing slow qubit frequency drifts and decoupling from the optical phase of the LS gate laser. Using this technique, the LS gate can also be implemented without the requirement to intrinsically null the differential light shift. This opens the opportunity to apply the gate to qubits with an energy difference in the optical domain, without careful selection of the laser frequency. Such gates were theoretically described^38^ and implemented^39^ for an optical qubit formed by sub-levels of the *S*_1/2_ and *D*_5/2_ manifold in ^40^Ca^+^ ions. We now describe how this gate scheme can be generalized to generate genuine qudit entanglement for qudits of arbitrary dimension encoded in the Zeeman sub-levels of the *S*_1/2_ and *D*_5/2_ manifolds of ^40^Ca^+^ ions, see Fig. 1. Formally, the two-ion LS Hamiltonian, after adiabatic elimination of the excited states and application of the Lamb-Dicke and rotating-wave approximations, can be written in the interaction frame of the ions’ center-of-mass (COM) motion as

1 $${H}_{{{{{{{{\rm{LS}}}}}}}}}=\frac{i\hslash \eta }{2}\mathop{\sum}\limits_{j}\mathop{\sum}\limits_{N}{{{\Delta }}}_{N,j}\left|\,{j}_{N}\right\rangle \left\langle \,{j}_{N}\right|{e}^{-i\delta t}{e}^{i{\varphi }_{N}}{a}^{{{{\dagger}}} }+{{{{{{{\rm{h.c.}}}}}}}}.$$

Here, Δ_*N*,*j*_ represents the light shift on the state $|\,j\rangle$ of ion *N*, *δ* the detuning from the motional mode frequency, *η* the Lamb-Dicke parameter, *a*^†^ the creation operator of the motional mode and *φ*_*N*_ the phase determined by the inter-ion distance within the traveling wave of the laser. Integrating Eq. (1), we obtain the propagator *U*_LS_(*t*) describing a state-dependent force on the ions’ motion that can be visualized as loops in motional phase space, which will periodically return to the origin after a time *t*_g_ = 2*π*/*δ*. During the evolution in phase space, each initial electronic state combination $|\,jk\rangle$ of the two ions will, in the most general case, pick up a different geometric phase *ϕ*_*jk*_ after a single LS gate pulse application.

**Fig. 3:**
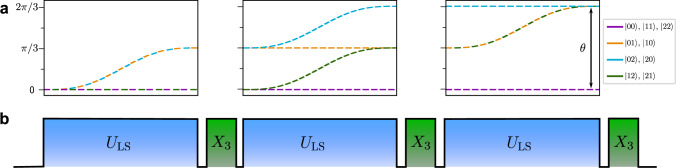
Application of the gate on two qutrits. **a** Phase evolution of the two-qutrit state components relative to the $|00\rangle$ ground state during the application of the LS gate pulses. States $|01\rangle$ and $|10\rangle$ are shown in orange, $|02\rangle$ and $|20\rangle$ in blue, and $|21\rangle$ and $|12\rangle$ in green. The equal electronic states $|00\rangle,|11\rangle,|22\rangle$ in purple do not acquire any relative phases. **b** Corresponding pulse scheme to implement the composite qudit light-shift gate operator *G*(*θ*) for a qutrit (*d* = 3). Light-shift gate pulses *U*_LS_(*t*_g_) are interlaced with cyclic permutation gates *X*_3_.

Similar to the qubit case, we can now symmetrize the geometric phases for qudits, while canceling differential light shifts. We achieve this by encoding our qudits in the $|0\rangle={S}_{1/2,{m}_{j}=-1/2}$ ground state and the Zeeman sub-levels of the *D*_5/2_ manifold as $|i\rangle$, with *i* ∈ {1, 2, 3, 4}, see Fig. 1. We then choose a wavelength close to the *S*_1/2_ ↔ *P*_1/2_ transition, which for our choice of encoding results in Δ_*N*,0_ ≫ Δ_*N*,*j*_ for *j* ≠ 0. This allows us to neglect differences in phase shifts between *D*_5/2_ levels induced by the LS gate pulse. We interleave *d* applications of *U*_LS_(*t*_g_) with two-ion cyclic permutations of the form ${X}_{d}=(\mathop{\sum }\nolimits_{j=0}^{d-1}|\,j+1\,({{{{{{{\rm{mod}}}}}}}}\,d)\rangle \langle \; j|)^{\otimes 2}$, where the populations of each level are transferred to the level with the next higher index. This ensures that each logical state spends an equal amount of time in each physical energy level. After application of the sequence $G={({X}_{d}{U}_{{{{{{{{\rm{LS}}}}}}}}}({t}_{{{{{{{{\rm{g}}}}}}}}}))}^{d}$ we find the phases ${\widetilde{\phi }}_{jk}$ imprinted on the states $|\,jk\rangle$

2 $$\widetilde\phi_{jk}=\left\{\begin{array}{cc}{\sum }_{k=0}^{d-1}{\phi }_{kk}\hfill\quad \quad &{{{{{{{\rm{if}}}}}}}}\,k=j \\ {\sum }_{k=0}^{d-1}{\sum }_{j < k}{\phi }_{jk}\quad &{{{{{{{\rm{else}}}}}}}},\hfill\end{array}\right.$$

where *ϕ*_*jk*_ refer to the phases from the constituent *U*_LS_(*t*_g_) pulses. For *d* = 2 the sequence ${({X}_{2}{U}_{{{{{{{{\rm{LS}}}}}}}}}({t}_{{{{{{{{\rm{g}}}}}}}}}))}^{2}$ corresponds to the standard qubit light-shift gate with spin echo^39^. Equation (2) shows that after symmetrization we are left with only two different phases, one for the cases where both ions are in the same state and one where the ions are in different states, see Fig. 3. Hence, up to a global phase, the qudit light-shift gate operation *G*(*θ*) can be described for all *d* by

3 $$G(\theta ):\left\{\begin{array}{ll}|\,jj\rangle \to |\,jj\rangle \hfill \quad \quad &\\ |\,jk \rangle \to \exp (i\theta )|\,jk \rangle \quad \quad &{{{{{{{\rm{if}}}}}}}}\,j\,\ne \,k.\end{array}\right.$$

The operator *G*(*θ*) directly generates genuine qudit entanglement as opposed to merely embedding qubit-level entanglement in a larger Hilbert space^17^. This will enable the generation of high-fidelity qudit entanglement with a single gate operation. In the experiment *θ* can be chosen freely by simultaneously varying the gate detuning *δ* and the gate time *t*_*g*_ or changing the laser power in order to adjust the light shifts Δ_*N*,*j*_. A more detailed derivation of Eq. (3) for the case of a qubit and qutrit is found in the supplementary note 1.

The gate is performed on two ^40^Ca^+^ ions, trapped 110 μm above the surface of a segmented surface Paul trap. The ion trap is mounted inside a liquid Helium flow-cryostat and is operated at ~ 35 K^40^. Static potentials applied to a set of DC electrodes confine the ions along the axial direction with an axial COM mode frequency of *ω*_COM_/2*π* ≈ 1.1 MHz. Radio-frequency potentials create radial confinement with frequencies {*ω*_*x*_, *ω*_*y*_}/2*π* ≈ {3.5 MHz, 3.2 MHz}. Outside the vacuum chamber, a pair of Helmholtz coils aligned 45^∘^ with respect to the axial trap direction generates a magnetic field of ~ 3.6 G, defining the quantization axis. The ${S}_{1/2,{m}_{j}=-1/2}$ ground state and the sub-levels of the *D*_5/2_ manifold are used for encoding the qudits, see Fig. 1. Single-qudit rotations between $|0\rangle$ and $|i\rangle$ are implemented using a 729 nm laser with a linewidth of ~10 Hz.

The interaction of Eq. (1) is generated by a pair of perpendicular laser beams, one of which is parallel and the other perpendicular to the applied magnetic field, each with a waist of approximately 45 μm, see Fig. 2. In this configuration, the difference wavevector of the two beams is parallel to the axial trap direction to only couple to the ions’ axial motion. The two beams are derived from a single frequency-doubled Titanium-Sapphire laser at a wavelength of *λ* ≈ 401.2 nm, approximately 8.1 THz red-detuned from the *S*_1/2_ ↔ *P*_1/2_ transition. This results in low scattering errors on the order of 10^−4^ for an LS gate pulse with a duration of *t*_g_ ~ 35 μs. The LS force on the state $|0\rangle$ is maximized by choosing both beams to be vertically polarized. The detuning between the beams is chosen as (*ω*_COM_ + *δ*) to couple primarily to the axial COM motion.

Maximizing the differential light shift for a given beam intensity requires the inter-ion distance to be an integer or half-integer multiple of the period of the traveling wave pattern created by the LS gate beams^36^. Imperfect spacing decreases the phase difference between equal and unequal states during the application of *U*_LS_ on the two ions, thus reducing the achievable gate speed for a given beam intensity. Since most error sources scale with the gate duration, correctly choosing the spacing is crucial for achieving low error rates. Experimentally, we adjust the inter-ion spacing by varying the voltages on the trap electrodes, which create the confinement in the axial direction.

The ion spacing is calibrated by initializing the ions in $|00\rangle$ and applying a resonant (*δ* = 0) LS gate pulse with variable time. If the spacing is set to a half-integer multiple of the standing wave, the breathing mode is excited to a coherent state, whereas the motion of the COM mode remains unaffected. For an integer spacing the relation between the motional modes is inverted. The motional state is read out by measuring the excitation when shelving the ions on the respective red sideband of the *S*_1/2_ ↔ *D*_5/2_ transition. By observing excitation of only the breathing mode, we infer that the inter-ion distance is set appropriately, and the unwanted phase accumulation of the equal states is minimized.

After Doppler cooling, the ions’ axial motional modes are cooled to around 0.1 quanta by resolved sideband cooling. We then initialize the ions in the $|00\rangle$ state via optical pumping. We create an equal superposition of all qudit states by applying the operator

4 $$P=\mathop{\prod }\limits_{j=1}^{d-1}{R}^{0,j}({\vartheta }_{j},0),$$

with rotation angle ${\vartheta }_{j}=2\arcsin (1/\sqrt{j+1})$ and

5 $${R}^{j,k}(\vartheta,\,\phi )=\exp \left(-i\frac{\vartheta }{2}\left({\sigma }_{1}^{j,k}(\phi )+{\sigma }_{2}^{j,k}(\phi )\right)\right)$$

where ${\sigma }_{N}^{j,k}(\phi )=\cos (\phi ){\sigma }_{x}^{j,k}+\sin (\phi ){\sigma }_{y}^{j,k}$ denotes the rotation on ion *N* on the transition $|\,j\rangle \leftrightarrow |k\rangle$ for Pauli matrices *σ*_*x*_,*σ*_*y*_. Each rotation *R*^*j*,*k*^(*ϑ*, *ϕ*) is implemented by a resonant 729 nm laser pulse, where *ϕ* is determined by the laser phase and *ϑ* by the pulse area. We then apply the sequence ${({X}_{d}{U}_{{{{{{{{\rm{LS}}}}}}}}}({t}_{{{{{{{{\rm{g}}}}}}}}}))}^{d}$, where the generalized spin echo *X*_*d*_ is implemented by a sequence of *π*-pulses on the $|0\rangle \leftrightarrow |\,j\rangle$ transitions. For *d* > 2 the phase of the first laser pulse of each *X*_*d*_ is shifted by *π* to decrease the errors due to over-rotation of the local pulses, leading to a sequence of

6 $${X}_{d}=\mathop{\prod }\limits_{j=1}^{d-1}{R}^{0,j}(\pi,\,{\delta }_{j1}\,\pi )$$

for the permutation operator, where *δ*_*ij*_ denotes the Kronecker delta. After the final permutation, we apply the conjugate *P*^†^ of the initial preparation sequence. Up to dimension *d* = 4, this leaves the system in a maximally entangled state of the form $|{{{\Psi }}}_{d}\rangle=\mathop{\sum }\nolimits_{j}^{d-1}|\,jj\rangle /\sqrt{d}$, whereas for *d* = 5 the sequence will result in the state $|{{{\Psi }}}_{5}\rangle=(3|00\rangle+2\mathop{\sum }\nolimits_{j=1}^{4}|\,jj\rangle )/5$. This is likely a consequence of there only being a single phase applied to all *d*^2^ − *d* components of the state. In order to generate maximal entanglement in higher dimensions it might thus be necessary to use multiple applications of the gate, or generalize the gate action such that it imparts different phase shifts to different state components.

We can directly estimate the state fidelity and the amount of entanglement of the generated states from the relative amplitudes of the components $|ii\rangle$, as well as their pairwise coherences. Experimentally, the population of the $|00\rangle$ state can be measured by driving the *S*_1/2_ ↔ *P*_1/2_ transition with a 397 nm laser and collecting the fluorescence photons on a photo-multiplier tube. Using additional *π*-pulses ${T}_{0}^{j}={R}^{0,j}(\pi,0)$, the same procedure gives access to all components $|\,jj\rangle$. The coherence terms between the states $|00\rangle$ and $|\,jj\rangle$ are estimated by applying a *π*/2-pulse ${A}_{0,\phi }^{j}={R}^{0,j}(\pi /2,\,\phi )$ with variable phase *ϕ* before performing the fluorescence readout. Applying ${T}_{0}^{k}$ before ${A}_{0,\phi }^{j}$ allows us to measure the coherence between the states $|kk\rangle$ and $|\,jj\rangle$. We then extract the coherence between the two terms from the parity oscillations by Bayesian parameter estimation, which accounts for measurement statistics and guarantees that the results stay physically possible.

**Fig. 4:**
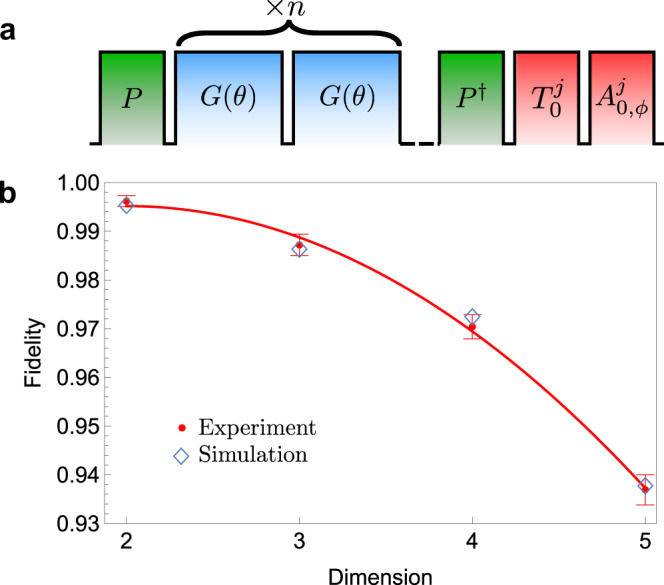
Fidelity decay measured using multiple gates. **a** Schematic of the measurement sequence. Two ions initialized in $\left|00\right\rangle$ are rotated into an equal superposition of all states by applying the operator *P* with the 729 nm laser. After applying the gate operator *G*(*θ*) a variable number of times *n* a reversed preparation pulse *P*^†^ is applied. The populations of the resulting state are measured by a set of transfer pulses ${T}_{0}^{j}$, which are resonant *π* pulses between $\left|0\right\rangle \leftrightarrow \left|\,j\right\rangle$ to transfer the state $\left|\,j\right\rangle$ to the *S*_1/2_ manifold, allowing us to distinguish the qudit states. An analysis pulse ${A}_{0,\phi }^{j}$ consisting of a resonant *π*/2 pulse between $\left|0\right\rangle \leftrightarrow \left|\,j\right\rangle$ with variable phase *ϕ* is used to measure the coherence between the $\left|0\right\rangle$ and $\left|\,j\right\rangle$ levels. Combined with the transfer pulses, all pairwise coherences can be measured. **b** A plot of qudit gate fidelity as a function of dimension. The average gate fidelities, shown as red circles, are extracted from fits to the fidelity decay when applying multiple gates *G*(*θ*) between *P* and *P*^†^. The error bars correspond to 1 standard deviation in the fit parameters. A quadratic curve has been fitted to the data to highlight the empirically observed scaling of the fidelity with dimension. The simulated fidelities from a detailed noise model are shown as blue diamonds, see supplementary note 2 for details.

The observed fidelity is affected by state-preparation-and-measurement (SPAM) errors, including the pulses *P*, *P*^†^ the transfer pulse ${T}_{0}^{j}$ and analysis pulse ${A}_{0,\phi }^{j}$. In order to separate the errors from SPAM and gate *G*(*θ*) for each dimension *d*, we insert up to 9 applications of *G*(*θ*) between the pulses *P* and *P*^†^ (see Fig. 4 (a)), and compute the state fidelity for each *n* that results in an entangled state. We then fit an exponential decay to estimate the fidelity of a single gate. Such repeated gate applications, however, are also sensitive to the presence of non-Markovian noise in our system that leads to deviations from purely exponential decay. The extracted fidelities should thus be interpreted as an estimate for the SPAM corrected average gate performance over a sequence of length *n*.

We apply this procedure for *d* = 2, 3, 4, 5 and obtain fidelities of 99.6(1)%, 98.7(2)%, 97.0(3)%, 93.7(3)%, respectively. While the intrinsic limits on gate fidelity due to finite state lifetime and Raman beam scattering depend only weakly on the dimension *d* (see supplemental note 2), the measured gate performance degrades quadratically with dimension as seen in Fig. 4 (b). This can be understood if the total gate error is dominated by technical noise sources that do not scale linearly with *d*. To investigate this we construct a numeric error model that computes the expected decay data using all independently measured error sources as inputs (see supplementary note 2), reproducing the observed data with very good agreement (blue diamonds in Fig. 4 (b)). This model suggests that while for *d* = 2 the gate fidelity is limited by the motional coherence time of the ion and frequency noise of the gate laser, for higher dimension the dominant error sources become the gate Rabi frequency noise and slow frequency noise that causes dephasing of the local operations. We can hence conclude that the gate fidelity in higher dimensions can be significantly improved if technical noise sources such as magnetic field noise contributing to the aforementioned local operation dephasing or Rabi frequency fluctuations can be suppressed.

**Fig. 5:**
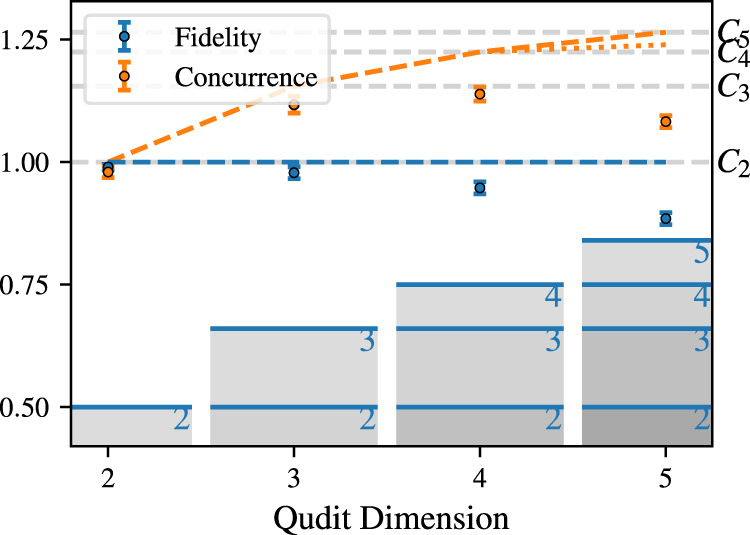
Generation of genuine qudit entanglement. The measured state fidelity for *d* = 2, 3, 4, 5 is shown as blue data points and the corresponding concurrence as orange squares. The ideal values for maximally entangled states (the experimental target states) are shown as dashed (dotted) lines. The gray shaded bars represent the lower bound on the fidelity (blue data points) for certifying maximal Schmidt number entanglement, while the gray horizontal lines indicate the concurrence *C*_*d*_ for a maximally entangled state in dimension *d*. Error bars correspond to one standard deviation of experimental shot noise.

We furthermore evaluate the entanglement properties^41^ of the states generated by a single application of the gate, including the Schmidt number, Concurrence, and Entanglement of Formation, see Fig. 5 and supplementary note 3 for details. We find that the concurrence for all states with *d* > 2 significantly exceeds the maximal possible value for any qubit state. Crucially, while the concurrence growth is expected to slow asymptotically with dimension, the Schmidt number in each dimension is maximal, indicating the presence of genuine qudit entanglement up to *d* = 5. The Schmidt number has also been suggested to play a crucial role in the computational complexity of a quantum system^42^.

## Discussion

We have demonstrated an experimental realization of a gate that directly generates native qudit entanglement between two trapped ions. The major difference between previously demonstrated qudit entangling schemes^17^ and our scheme is that the gate natively couples to all transitions, whereby it creates entanglement between the multiple qudit levels in a single application of the gate rather than through repeated applications of qubit entangling operations that couple to individual transitions and thereby only generate two-level entanglement in each step. This fundamental difference dictates not only the kind of entangling dynamics that can be realized, but also the error contributions and requirements on the experimental control. As a result the calibration of our native qudit gate compares favorably to multiple applications of a pairwise entangling Mølmer-Sørensen gate. In the latter case, the gate requires careful adjustment of the entangling laser control parameters for each of the desired $|S\rangle \leftrightarrow |D\rangle$ transitions including compensation for light shifts and induces undesired phase shifts on spectator levels, which have to be tracked. For our qudit phase gate, increasing the dimensionality of the entangling space just requires one extra local operation per additional *D*_5/2_ sub-level to be calibrated and the power of the gate laser to be adjusted by a known analytical ratio that is not sensitive to qubit frequency shifts from light shifts or otherwise, since the gate beam is far off-resonant. Interestingly, while all demonstrated schemes for creating genuine qudit entanglement exhibit a linear increase in gate duration with qudit dimension, this may be overcome for some interactions at the cost of a linear increase in control parameters^43^. Moreover, while we demonstrated a highly symmetrized version of the gate, exploiting different light shifts on different ground- and excited state levels allows for a wide range of gate actions, accessible through local operations alone.

## Supplementary information

Supplementary Information

Peer Review File

## Data Availability

The data generated in this study is deposited on Zenodo at 10.5281/zenodo.7688595.

## Code availability

The code used for simulation of the error budget is available upon request.
