# Supplementary material for: Native qudit entanglement in a trapped ion quantum processor
Source: Nat Commun. 2023 Apr 19;14:2242. doi: 10.1038/s41467-023-37375-2 (PMC10115791; doi:10.1038/s41467-023-37375-2)
Supplement: Supplementary file 1 — Supplementary Information [file 41467_2023_37375_MOESM1_ESM.pdf]

Supplementary Information
Native qudit entanglement in a trapped ion quantum processor

SUPPLEMENTARY NOTE 1 - GATE ACTION IN THE CASE OF THE QUBIT AND QUTRIT

In this section we explicitly show how the phase acquisition in two and three dimensions leads to entanglement. For a qubit, the gate acts as follows:

$$\begin{aligned}
(X_2 U_{\mathrm{LS}}(t_{\mathrm{g}}))^2 \left|00\right\rangle &\to \exp(\phi_{00} + \phi_{11}) \left|00\right\rangle \\
(X_2 U_{\mathrm{LS}}(t_{\mathrm{g}}))^2 \left|01\right\rangle &\to \exp(\phi_{01} + \phi_{10}) \left|01\right\rangle \\
(X_2 U_{\mathrm{LS}}(t_{\mathrm{g}}))^2 \left|10\right\rangle &\to \exp(\phi_{10} + \phi_{01}) \left|10\right\rangle \\
(X_2 U_{\mathrm{LS}}(t_{\mathrm{g}}))^2 \left|11\right\rangle &\to \exp(\phi_{11} + \phi_{00}) \left|11\right\rangle ,
\end{aligned}$$

with

$$\phi_{jk} = \frac{\pi\eta^2(\Delta_{0,j}e^{i\varphi_0} + \Delta_{1,k}e^{i\varphi_1})^2}{2\delta^2}. \tag{1}$$

By choosing the ion spacing appropriately as described in the main text, the spatial phase of ion N can be described as $\varphi_N = N\pi$. With the additional assumption that the ions are equally illuminated within the travelling wave, we find $\Delta_{0,j} = \Delta_{1,j}$ for all j and therefore $\phi_{00} = \phi_{11} = 0$. The asymmetric states thus acquire a phase relative to the symmetric states. An equal superposition of all four states is mapped to a maximally entangled state when the condition $\phi_{01} = \phi_{10} = \pi/4$ is fulfilled. This can, for example, be achieved by changing the coupling strength $\Delta_{n,j}$ through changing the power of the laser beams, or by changing the detuning from the motional mode δ_{g}. It should be noted that the condition for the spatial phase is not a strict requirement, as a maximally entangling gate is still possible as long as $\varphi_n \neq N\pi/2$, albeit a higher laser power is required to compensate for the phase acquisition of ϕ_{00} and ϕ_{11}.

For the case of a qutrit we find

$$\begin{aligned}
(X_3 U_{\mathrm{LS}}(t_{\mathrm{g}}))^3 \left|00\right\rangle &\to \exp(\phi_{22} + \phi_{11} + \phi_{00}) \left|00\right\rangle \\
(X_3 U_{\mathrm{LS}}(t_{\mathrm{g}}))^3 \left|01\right\rangle &\to \exp(\phi_{20} + \phi_{12} + \phi_{01}) \left|01\right\rangle \\
(X_3 U_{\mathrm{LS}}(t_{\mathrm{g}}))^3 \left|10\right\rangle &\to \exp(\phi_{02} + \phi_{21} + \phi_{10}) \left|10\right\rangle \\
(X_3 U_{\mathrm{LS}}(t_{\mathrm{g}}))^3 \left|11\right\rangle &\to \exp(\phi_{00} + \phi_{22} + \phi_{11}) \left|11\right\rangle \\
(X_3 U_{\mathrm{LS}}(t_{\mathrm{g}}))^3 \left|12\right\rangle &\to \exp(\phi_{01} + \phi_{20} + \phi_{12}) \left|12\right\rangle \\
(X_3 U_{\mathrm{LS}}(t_{\mathrm{g}}))^3 \left|20\right\rangle &\to \exp(\phi_{12} + \phi_{01} + \phi_{20}) \left|20\right\rangle \\
(X_3 U_{\mathrm{LS}}(t_{\mathrm{g}}))^3 \left|21\right\rangle &\to \exp(\phi_{10} + \phi_{02} + \phi_{21}) \left|21\right\rangle \\
(X_3 U_{\mathrm{LS}}(t_{\mathrm{g}}))^3 \left|22\right\rangle &\to \exp(\phi_{11} + \phi_{00} + \phi_{22}) \left|22\right\rangle .
\end{aligned}$$

If we again make the assumption that the ions are equally illuminated, we find $\phi_{jk} = \phi_{kj}$ for all j, k. It then becomes evident that the states $\left|00\right\rangle, \left|11\right\rangle, \left|22\right\rangle$ acquire a relative phase compared to all the other states. Genuine multipartite entanglement is obtained if $\phi_{01} + \phi_{02} + \phi_{12} = 2\pi/3$. The phase acquisition during the gate is graphically illustrated in Fig. 3 of the main text.

SUPPLEMENTARY NOTE 2 - ERROR MODEL

The gate error analysis is based on numerically integrating the Lindblad master equation with collapse operators that describe motional heating and motional dephasing. All other noise sources (with the exception of the local gate laser) are assumed to be slow on the time scale of one experiment and thus are treated as static offsets sampled from a normal distribution with standard deviations listed in table 1 and averaged over 100 runs of the numerical integration. All input values are measured independently using the ions as probes with appropriate techniques (e.g. sideband thermometry, Ramsey spectroscopy, Rabi spectroscopy, Stark shift measurements etc.). When analysing the 729 laser intensity noise, we discovered further pulse area variations between subsequent π pulses on timescales comparable to those presented during the gate operation. We thus include an additional *fast* noise parameter that samples a new Rabi frequency for each subsequent local pulse during a single gate simulation run. The frequency noise for the local operations contains a contribution from both the finite 729 nm laser linewidth and magnetic field noise. Since each level in our qudit has a different magnetic field sensitivity, we scale the value in table 1 by empirically measured coherence times on the different transitions.

Since the action of these slow noise components is non-Markovian we will observe a different error per gate depending on how many gates we apply in succession. To be consistent with the decay fit method that we use to correct for SPAM and extract the fidelity from the experimental data, all simulation fidelities are extracted using an exponential fit to the same amount of gates as applied in figure 4b of the main text.

The intrinsic two-qubit gate fidelity due to scattering and state decay can be calculated analytically following the analysis in [1]. We additionally extend the D state error to be determined not just by the gate length, but also the total time spent applying our generalised spin echo local operations. Extending this analysis to higher dimensions can be significantly simplified since the contributions to the scattering error arising from scattering (both elastic and inelastic) from the D states are negligible. We thus obtain the $d > 2$ errors by scaling the qubit error linearly by the appropriate gate time and laser intensity and correct for the fraction of the initial superposition state present in the S state for $d > 2$ relative to the qubit.

Error source	Error value	Unit	Infidelity $d=2$	$d=3$	$d=4$	$d=5$
Motional heating rate	15	ph/s	1.3×10^{-4}	2.2×10^{-4}	4×10^{-4}	7×10^{-4}
Motional coherence	16	ms	1.2×10^{-3}	2.2×10^{-3}	5×10^{-3}	5×10^{-3}
Motional mode occupation	0.1	ph	2×10^{-4}	3×10^{-4}	4×10^{-4}	1.3×10^{-3}
Gate Rabi frequency	1	%	2.2×10^{-4}	4×10^{-3}	8×10^{-3}	7×10^{-3}
Slow local Rabi frequency	0.6	%	3×10^{-4}	4×10^{-4}	6×10^{-4}	3×10^{-3}
Fast local Rabi frequency	0.7	%	2.5×10^{-4}	1×10^{-3}	1.4×10^{-3}	1.6×10^{-3}
Local Rabi imbalance	1	%	4×10^{-4}	6×10^{-4}	1.5×10^{-3}	6×10^{-3}
Gate laser frequency noise	$2\pi \times 200$	rad/s	1.3×10^{-3}	1.8×10^{-3}	3×10^{-3}	3×10^{-3}
Local operation frequency noise	$2\pi \times 19$	rad/s	1.5×10^{-6}	2.3×10^{-3}	6×10^{-3}	3.3×10^{-2}
Elastic & inelastic scattering			1.6×10^{-4}	2×10^{-4}	3×10^{-4}	3×10^{-4}
$D_{5/2}$ state decay	1	s	8×10^{-5}	2×10^{-4}	5×10^{-4}	9×10^{-4}
Total			4.2×10^{-3}	1.3×10^{-2}	2.7×10^{-2}	6.2×10^{-2}

Supplementary Table 1: Error sources and the corresponding simulated infidelity for the gate in qudit dimension $d = 2, 3, 4, 5$ based on measured noise parameters.

SUPPLEMENTARY NOTE 3 - COMPUTING QUDIT ENTANGLEMENT

We use two figures of merit to evaluate the entangling properties of the new gate. The first is the Schmidt number, also often referred to as dimensionality of entanglement [2]. It quantifies the minimal local dimension needed to express the correlations present in the state. Formally, consider a bipartite state between parties A and B, then the Schmidt rank is

$$r(\rho) := \inf_{\mathcal{D}(\rho)} \max_{|\psi_i\rangle \in \mathcal{D}(\rho)} \text{rank}(\text{Tr}_B |\psi_i\rangle\langle\psi_i|) \,, \tag{2}$$

where $\mathcal{D}[\rho]$ refers to the set of all decompositions, i.e. all pairs of probabilities and pure state vectors that add up to the whole state: $\mathcal{D}[\rho] := \{(p_i, |\psi_i\rangle) : \sum_i p_i |\psi_i\rangle\langle\psi_i| = \rho\}$. While computing the Schmidt number is at least as hard as deciding whether a given density matrix is separable (NP-hard), there are easily computable lower bounds. In particular, the fidelity of an experimental state ρ_{exp}, with an entangled target state

$$|\psi_T\rangle = \sum_{i=0}^{d-1} \lambda_i |ii\rangle \,, \tag{3}$$

is bounded by its Schmidt number r through $F(\rho_{exp}, |\psi_T\rangle\langle\psi_T|) \leq \sum_{i=0}^{r-1} \lambda_i^2$, with the Schmidt coefficients λ_i sorted in order of decreasing absolute value. The ideal states generated by the gate are the maximally entangled state in $d = 2, 3, 4$ and the state $|\psi_T\rangle = 0.6\,|00\rangle + 0.4(|11\rangle + |22\rangle + |33\rangle + |44\rangle)$ in $d = 5$. With respect to these target states, we obtain fidelities of $99.0 \pm 0.6\%, 97.8 \pm 1.2\%, 94.7 \pm 1.2\%, 88.5 \pm 1.2\%$, thus proving that the Schmidt number is indeed maximal in all experimentally implemented dimensions.

The second figure of merit is a smooth quantifier that is related to the entanglement cost. The entanglement of formation is a generalisation of the entanglement entropy for pure states as the average entanglement entropy (=entropy of the reduced density matrix) of a pure state decomposition of a density matrix, minimised over all possible decompositions. Its regularisation is the exact entanglement cost, i.e. the average number of maximally entangled states needed per copy of the system state to create it via local operations and classical communication (LOCC) in the asymptotic limit of infinitely many copies.

$$E_{oF} := \inf_{\mathcal{D}(\rho)} \sum_i p_i S((\text{Tr}_B |\psi_i\rangle\langle\psi_i|)) \,, \tag{4}$$

where S is the von Neumann entropy. It can be lower bounded in various ways, the most easily accessible in a scalable manner is through the same elements that were needed to compute fidelity. Those can be used to compute a lower bound on the Renyi-2-entropy of entanglement [3]. These lower bounds, however, are known to already have some drawback when computed for the pure target states. Here, the Renyi-2-entropy is already different from the von Neumann entropy as soon as one departs from the maximally entangled state as target state (as it is in our case of dimension 5). So generally, the actual entanglement of formation will be quite seriously underestimated. Nonetheless, despite the lower bound, the non-sharpness and all experimental imperfections, the entanglement of formation still is above anything even the most perfect qubit could achieve, solidifying also the asymptotic entanglement properties of our qudit phase gate.

The concurrence is a convenient quantifier of entanglement that can be defined as the convex roof extension of the square-root of the linear entropy.

$$C := \inf_{\mathcal{D}[\rho]} \sum_i p_i \sqrt{2(1 - \text{Tr}[\text{Tr}_B(|\psi_i^{AB}\rangle\langle\psi_i^{AB}|)^2]} \,. \tag{5}$$

This quantity is clearly related to the Renyi-2-entropy of entanglement $S_2(|\psi_i^{AB}\rangle\langle\psi_i^{AB}|) := -\log(\text{Tr}_B(|\psi_i^{AB}\rangle\langle\psi_i^{AB}|)^2)$, which in turn lower-bounds the von Neumann entropy used in the entanglement of formation. While it is NP-hard to determine the concurrence in high-dimensional systems exactly, even if the density matrix is completely known, it can luckily be lower-bounded by easily accessible measurements. In particular, the lower bound from Ref. [3] is given by

$$C \geq \frac{1}{\sqrt{d(d-1)}} \sum_{i \neq j} \langle ii|\rho|jj\rangle - \sqrt{\langle ij|\rho|ij\rangle\langle ji|\rho|ji\rangle} \,, \tag{6}$$

	$d = 2$	$d = 3$	$d = 4$	$d = 5$
Fidelity	0.990 ± 0.006	0.978 ± 0.012	0.947 ± 0.012	0.885 ± 0.012
Fidelity Threshold	0.5	0.66	0.75	0.84
Concurrence $\geq$	0.98 ± 0.01	1.12 ± 0.02	1.14 ± 0.01	1.08 ± 0.01
Max. Concurrence	1	1.154	1.224	1.264
Entanglement of Formation $\geq$	0.97 ± 0.08	1.50 ± 0.12	1.61 ± 0.13	1.26 ± 0.06
Schmidt number	2	3	4	5

Supplementary Table 2: Fidelities and entanglement measures for the states produced by a single entangling gate for qudit dimensions $d = 2, 3, 4, 5$. The fidelity threshold corresponds to the minimum fidelity to achieve maximal Schmidt number, while the maximum concurrence gives the value for a pure maximally entangled state of a given dimension.

where the individual density matrix elements $\langle ij|\rho|ij\rangle$ can be estimated from measurements in the computational basis and the remaining off-diagonal elements through pairwise interference measurements.

[1] B. C. Sawyer and K. R. Brown, Phys. Rev. A **103**, 022427 (2021).
[2] N. Friis, G. Vitagliano, M. Malik, and M. Huber, Nature Reviews Physics **1**, 72 (2019).
[3] M. Huber, M. Perarnau-Llobet, and J. I. de Vicente, Phys. Rev. A **88**, 042328 (2013).
