# Supplementary material for: Native qudit entanglement in a trapped ion quantum processor
Source: Nat Commun. 2023 Apr 19;14:2242. doi: 10.1038/s41467-023-37375-2 (PMC10115791; doi:10.1038/s41467-023-37375-2)
Supplement: Supplementary file 2 — Peer Review File [file 41467_2023_37375_MOESM2_ESM.pdf]

nature portfolio

Peer Review File

Native qudit entanglement in a trapped ion quantum processor

Open Access This file is licensed under a Creative Commons Attribution 4.0 International License, which permits use, sharing, adaptation, distribution and reproduction in any medium or format, as long as you give appropriate credit to the original author(s) and the source, provide a link to the Creative Commons license, and indicate if changes were made. In the cases where the authors are anonymous, such as is the case for the reports of anonymous peer reviewers, author attribution should be to 'Anonymous Referee' followed by a clear attribution to the source work. The images or other third party material in this file are included in the article's Creative Commons license, unless indicated otherwise in a credit line to the material. If material is not included in the article's Creative Commons license and your intended use is not permitted by statutory regulation or exceeds the permitted use, you will need to obtain permission directly from the copyright holder. To view a copy of this license, visit http://creativecommons.org/licenses/by/4.0/.

Editorial Note: This manuscript has been previously reviewed at another journal that is not operating a transparent peer review scheme. This document only contains reviewer comments and rebuttal letters for versions considered at *Nature Communications*.

REVIEWERS' COMMENTS

Reviewer #1 (Remarks to the Author):

I read the revised manuscript and the authors' reply, and I believe the authors have satisfactorily addressed the previous comments. With the transfer, I can recommend its publication in Nature Communications.

Reviewer #2 (Remarks to the Author):

The authors have carefully and thoroughly responded to comments from both reviews given for their earlier submission, and revised the manuscript accordingly. I have no further comments on the manuscript.

The paper extends previous control of qudits to demonstrate two-qudit entanglement that generates entanglement between >2 states in each qudit, and this is a significant achievement. The paper includes appropriate information and references to assess the significance of such techniques for quantum computation/simulation, and I recommend publication in Nature Communications.
